# High ANO1 expression is a prognostic factor and correlated with an immunosuppressive tumor microenvironment in pancreatic cancer

**DOI:** 10.3389/fimmu.2024.1341209

**Published:** 2024-01-30

**Authors:** Guangnian Zhang, Zhihui Shu, Jun Yu, Jianshui Li, Pengsheng Yi, Bin Wu, Dawei Deng, Shu Yan, Yong Li, Dongmei Ren, Yifu Hou, Chuan Lan

**Affiliations:** ^1^Department of Hepatobiliary Surgery and Center of Severe Acute Pancreatitis, The Affiliated Hospital of North Sichuan Medical College, Nanchong, China; ^2^Department of Organ Transplantation, Sichuan Provincial People’s Hospital, University of Electronic Science and Technology of China, Chengdu, China; ^3^Clinical Immunology Translational Medicine Key Laboratory of Sichuan Province & Organ Transplantation Center, Sichuan Provincial People’s Hospital, University of Electronic Science and Technology of China, Chengdu, China

**Keywords:** aminooctylamine, prognostic factor, cancer-associated fibroblasts, tumor-infiltrating lymphocytes, tumor microenvironment, pancreatic cancer

## Abstract

**Background:**

Aminooctylamine (ANO1) plays an oncogenic role in various cancers. However. its role in pancreatic cancer (PC) has rarely been studied. This study investigated the prognostic value of ANO1 and its correlation with the tumor microenvironment (TME) in PC.

**Methods:**

Consecutive patients with PC (n = 119) were enrolled. The expression of ANO1 in cancer cells, the expression of fibroblast activation protein (FAP) and alpha smooth muscle actin in cancer-associated fibroblasts (CAFs), and the numbers of CD8- and FOXP3-positive tumor-infiltrating lymphocytes (TILs) were evaluated using immunohistochemistry. The prognostic value of ANO1 and its correlation with CAF subgroups and TILs were analyzed. The possible mechanism of ANO1 in the TME of PC was predicted using the the Cancer Genome Atlas (TCGA) dataset.

**Results:**

The expression of AN01 was correlated with overall survival (OS) and disease-free survival. Multi-factor analysis showed that high ANO1 expression was an independent adverse prognostic factor for OS (hazard ratio, 4.137; P = 0.001). ANO1 expression was positively correlated with the expression of FAP in CAFs (P < 0.001) and negatively correlated with the number of CD8-positive TILs (P = 0.005), which was also validated by bioinformatics analysis in the TCGA dataset. Moreover, bioinformatic analysis of the TCGA dataset revealed that ANO1 may induce an immunosuppressive tumor microenvironment in pancreatic cancer in a paracrine manner.

**Conclusion:**

ANO1 is a prognostic factor in patients with PC after radical resection. ANO1 may induce an immunosuppressive tumor microenvironment in PC in a paracrine manner, suggesting that ANO1 may be a novel therapeutic target.

## 1 Introduction

Pancreatic cancer (PC) ranks the third leading cause of cancer-related death in the developed countries and is expected to be the second-leading cause of cancer-related mortality by 2040 (1, 2). Interest in PC has increased owing to its increasing annual incidence and high mortality (3). Currently, surgery is the main curative treatment for patients with PC. However, less than 20% of patients are suitable for operation at diagnosis (4). Furthermore, even in cases where surgery is suitable, about 75% of them will experience recurrence within 2 years (5), and the 5-year overall survival (OS) rate remains only less than 10% (6). For patients with recurrent or advanced tumors, other treatments, including chemotherapy, radiotherapy, and immune therapies, have shown limited efficacy. Therefore, it is necessary to determine and implement the new treatment strategies that benefit more patients.

Aminooctylamine (ANO1) is a calcium-activated chloride channel protein (7) which serves the functions such as smooth muscle contraction, the epithelial ion transport and glandular secretion (8, 9). In studies to date that have shown that ANO1 is overexpressed in multiple diseases and tumor types and contributes to the process of carcinogenesis (10). For example, in esophageal squamous cell carcinoma, both mRNA and protein levels of ANO1 were upregulated and correlated with an unfavorable clinical prognosis (11). Additionally, ANO1 is highly expressed in breast, ovarian, and hepatocellular carcinomas and promotes the malignant biological behavior of cancer cells (12–14). Although one research showed that ANO1 was overexpressed in PC (15), the prognostic value and specific role of ANO1 in PC progression remain unclear, and its association with the tumor microenvironment (TME) has not been explored.

TME play a crucial role in regulating cancer cells progression. This topic has received increasing appreciation recently. The pancreatic TME contains multiple fibroblasts and heterogeneous immune cell populations with inhibitory effects (16). Cancer-associated fibroblasts (CAFs) are a heterogeneous group consisting of myofibroblast CAFs (myCAFs), inflammatory CAFs (iCAFs), and antigen-presenting CAFs (17). MyCAFs are located near tumor cells and is considered to be the cause of the extracellular matrix deposition. iCAFs tend to dominate the space between islands of PC cells and release inflammatory cytokines such as interleukin-6 (IL-6) (18). Fibroblast activation protein (FAP) and alpha smooth muscle actin (α-SMA) are markers of myCAFs and iCAFs, respectively. Recent studies have shown that targeted FAP-positive CAFs may inhibit tumor growth (19–21). In addition, the absence of FAP in the stromal cells delayed the progression of PC disease in mice (19). α-SMA-positive CAFs have tumor-inhibiting effects in a genetically engineered mouse model of PC and contribute to the differentiate of tumor-infiltrating T cells (22). Positive α-SMA was associated with increased OS, whereas positive FAP was associated with decreased OS (23). Tumor-infiltrating lymphocytes (TILs) represent the immune response to tumor progression, and they are another important component of TME (24). CD8 and FOXP3 play important but opposite roles in cancer and are two specific subgroups of TILs (21). CD8-positive TILs are key immune cells in tumor immunity that kill cancer cells by triggering apoptosis, while foxp3 positive TILs are regulatory T cells (Tregs) with immunosuppressive functions that inhibit the proliferation and activation of CD8-positive TILs (25). A recent study found that IL-6 secreted by α-SMA positive CAF conferred chemotherapy resistance and negatively regulated T cells in the TME (26). In addition, growth factors and inflammatory cytokines secreted by CAFs activate oncogenic pathways in cancer cells. Interestingly, IL-6 and epidermal growth factor promote the expression of ANO1 by signal transducer and transcriptional activator 3 and phosphatidylinositol 3 kinase/protein kinase B signaling (27). This suggests that ANO1 levels can be regulated in cancer cells by cytokines, which may be the molecular mechanism by which ANO1 is involved in inflammation and tumor immunity. However, whether ANO1 regulates TIL and CAF populations in PC is unclear and requires further investigation.

Therefore, the aims of this study were to probe into the prognostic value of ANO1 in PC patients and explore the relationship between ANO1 expression and TIL and CAF populations.

## 2 Materials and methods

### 2.1 Patients and specimens

A total of 119 patients with pathologically confirmed PC who underwent surgery in the Affiliated Hospital of North Sichuan Medical College from January 2016 to June 2022 were included in this single-center study. None of the participants had received radiotherapy or chemotherapy before surgery, and their clinical data were complete. Fluorouracil or gemcitabine are mainly used as adjuvant chemotherapy. The pathological classification of pancreatic cancer was based on the 2019 WHO classification, and TNM staging is based on the American Joint Committee on Cancer 8th edition of the cancer staging system.

Clinical examinations and enhanced computed tomography were performed at 3-month intervals after the initial surgery for primary PC. Disease-free survival (DFS) was defined as the time from surgery to tumor recurrence or death. OS was defined as the time from surgery to death.

Written informed consent was obtained from all the patients, and the research program was approved by the Institutional Review Committee of the Affiliated Hospital of North Sichuan Medical College (number: 2023ER041-1). We adhered to the ethical standards of the relevant local and national human experimentation committees, as well as the 1964 Declaration of Helsinki and its subsequent amendments.

### 2.2 Immunohistochemistry and evaluation of TILs and CAFs

Paraffin-embedded sliced of PC patients were dewaxed in xylenes and ethanol and autoclaved in the fluid of the antigen repair (MVS-0100; Maixin biotechnology; Fuzhou; China) for 15 min to repair epitope of antigen. Immunohistochemical staining was performed using a ready-to-use immunohistochemical UltraSensitive SP reagent (mouse/rabbit) (KIT-9710; Maixin biotechnology; Fuzhou; China). Endogenous peroxidase activity was interdicted using an endogenous peroxidase blocker (reagent 1) for 10 min at room temperature and a non-specific stain blocker (reagent 2) for 10 minutes at room temperature. For CD8 and FOXP3 dyeing, we adopt rabbit polyclonal anti-CD8 (1:200 dilution; AF5126; Affinity Biosciences; Jiangsu; China) and anti-FOXP3 (1:200 dilution; AF6544; Affinity Biosciences; Jiangsu; China) antibodies, respectively. For FAP and α-SMA staining, we adopt anti-FAP (1:50 dilution; AF5344; Affinity Biosciences; Jiangsu; China) and anti-α-SMA (1:100 dilution; AF1032; Affinity Biosciences; Jiangsu; China) antibodies, respectively. Section of tissue were incubated with the primary antibody at 4°C overnight. They were then incubated with biotin-labeled sheep anti-mouse/rabbit IgG polymer (reagent 3) for 10 min at room temperature, Streptomyces anti-biotin protein-peroxidase (reagent 4) for 10 min at room temperature, and DAB (DAB-0031; Maixin biotechnology; Fuzhou; China) solution for color development for 1-3 min. Slides were then counterstained with hematoxylin. Two researchers ignored the clinicopathological data and evaluated the stained slides using a ×200 light microscope. For CD8 and FOXP3 staining, sequential slices of tumor sections of each patient was evaluated. The number of positive cells in a field 1 mm in diameter were counted in three fields containing a large number of cancer cells, and the results were expressed as the average of the counts in triplicate (number of cells per field). The one-way scoring system based on the dyeing strength of FAP and alpha SMA in CAFs expression for immunohistochemical analysis. The intensity of staining was divided into 0(no staining), 1(weak staining), 2(moderate staining), and 3(strong staining). Intensity of Staining of 0 or 1 was defined as the negative expression of FAP and α-SMA in CAFs, and staining intensity of 2 or 3 was defined as positive expression of FAP and α-SMA in CAFs.

### 2.3 Immunohistochemistry and evaluation of ANO1 positivity

Sample handling and immunohistochemistry were performed as mentioned above. After blocking endogenous peroxidase activity, samples were incubated with diluted ANO1 antibody (1:100 dilution; DF7769; Affinity Biosciences; Jiangsu; China) overnight, and assays were performed using reagents 3 and 4. A DAB color solution was used to observe a positive reaction, and slides were counterstained with hematoxylin. All staining results were scored independently by two researchers ignored the clinicopathological data as described above. Intensity of staining was rated to negative, weak, moderate, and strong expression, respectively. Negative or weak ANO1 expression was regarded as negative staining, and moderate or strong ANO1 expression was regarded as positive staining.

### 2.4 Bioinformatics and survival analyses

GEPIA was used to obtain the OS and DFS survival plots of ANO1 from the TCGA dataset. We used 50% as a cutoff value. A log-rank test was used to assess difference in survival between patients with high and low ANO1 expression. Hazard ratios and p-values were calculated using univariate Cox regression analysis.

### 2.5 Single sample gene set enrichment analysis and calculation of the ImmuneScore and StromalScore

Tumor-infiltrating immune cells were quantified by ssGSEA using the GSVA package in R. ssGSEA applies the gene signatures expressed by immune cell populations to individual cancer samples. The deconvolution approach used in this study included CD8-positive T cells and Tregs. We used the ssGSEA algorithm to explore the relationship between ANO1 expression and the infiltration of CD8-positive T cells and Tregs based on the PC dataset in TCGA (Firehose Legacy). The correlation between ANO1 and tumor-infiltrating immune cells was assessed using Spearman’s test.

After selecting the samples, we extracted the expression matrix from the samples and calculated the immune purity of the expression matrix using the “estimate” R package. We performed ssGSEA for each sample, and the immune infiltration (ImmuneScore) and overall stromal content (StromalScore) were calculated using the ESTIMATE algorithm.

### 2.6 Selection of differentially expressed genes

The patients were divided into high- and low-score groups based on the cutoff value of 50%. The selection of DEGs was performed according to the published method using the “edgeR” R package with P <0.05 and |logFC| >1. A volcano plot was used to visualize the DEGs. Network enrichment analysis was performed using gene set enrichment analysis (http://software.broadinstitute.org/gsea/index.jsp) to identify the possible pathways affected by ANO1. Moreover, a heatmap was generated to describe the upregulated or downregulated intersection genes of DEGs in pathways using a website tool (http://bioinformatics.psb.ugent.be/webtools/Venn/).

### 2.7 Statistical analyses

Continuous variables that conformed to a normal distribution are represented by the mean ± standard deviation, and continuous variables that did not conform to a normal distribution are represented by the median (upper quartile-lower quartile). The significance of differences was assessed using Student’s t-test or the Mann-Whitney U test. Differences in categorical variables were evaluated using the chi-squared test or Fisher’s exact test, as appropriate. Predictors of prognosis were determined using Cox proportional hazards regression analysis. Clinicopathological factors were analyzed by multivariable analysis, which included variables with P < 0.05 in the univariate analysis. The Kaplan–Meier method was used to estimate OS and DFS, and the log-rank test was used to compare survival curves. The Wilcoxon rank sum test and chi-square test were used for correlation analysis. P < 0.05 was considered statistically significant. All tests were performed using IBM SPSS Statistics 19 software.

## 3 Results

### 3.1 Patient characteristics

There were about 119 patients with PC who underwent surgical resection in all were enrolled in this study Among them, 73 (61.3%) experienced tumor recurrence and 90 (75.6%) died. The median follow-up period was 37 months.

**Figure 1:**
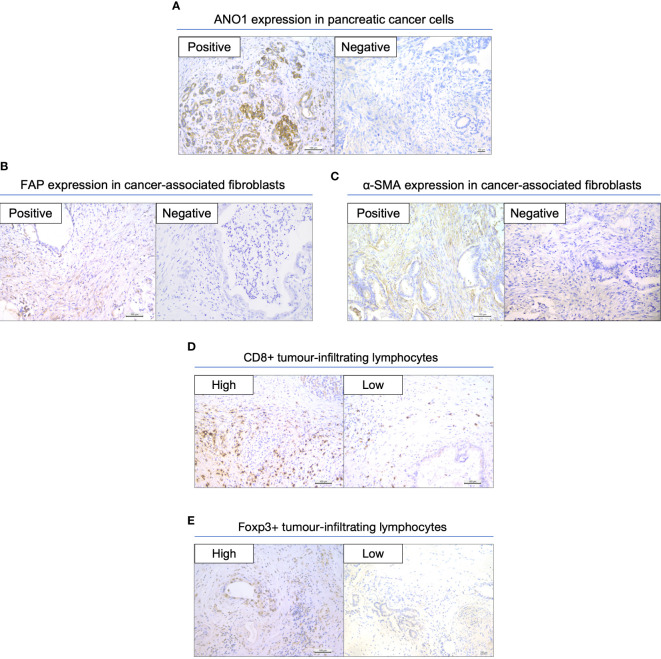
Immunohistochemical staining in resected pancreatic cancer specimens. **(A)** Expression of ANO1 in cancer cells. **(B)** Expression of FAP in CAFs. **(C)** Expression of α-SMA in CAFs. **(D)** Expression of CD8 in TILs. **(E)** Expression of FOXP3 in TILs. Scale bar = 100 µm. ANO1, aminooctylamine; FAP, fibroblast activation protein; CAF, cancer-associated fibroblast; α-SMA, alpha smooth muscle actin; TIL, tumor-infiltrating lymphocyte; FOXP3, forkhead box protein 3.

We assessed the expression of ANO1 in tissue samples from PC patients; 74 patients had positive ANO1 expression and 45 had negative ANO1 expression (**Figure 1A**). FAP and α-SMA expression in the CAFs was also assessed (**Figures 1B, C**). There were 74 patients with FAP positivity and 45 with FAP negativity, whereas there were 57 patients with α-SMA positivity and 62 with α-SMA negativity. CD8-positive TILs and FOXP3-positive TILs were also counted, and patients were defined as having high or low expression according to the 50% cutoff value (**Figures 1D, E**).

### 3.2 ANO1 expression was an independent prognostic factor for patients with pancreatic cancer

**Table 1** sum up the clinicopathological features of the ANO1-positive and ANO1-negative groups. There were no obvious differences in age, sex, total bilirubin level, CA19-9 level, tumor size, tumor differentiation, pT, pN, nerve invasion, and tumor location between ano1 positive and ano1 negative groups.

**Table 1:** Clinicopathological features based on ANO1 expression.

Characteristic	Total (n = 119)	ANO1 expression		p value
		Negative (n = 45)	Positive (n = 74)	
Age (y)	64.2 ± 9.4	63.6 ± 8.3	64.5 ± 10.1	0.449
Sex				0.550
Male	78 (65.5%)	31 (68.9%)	47 (63.5%)	
Female	41 (34.5%)	14 (31.1%)	27 (36.5%)	
CA19-9 (ng/mL)	216.9 (48.9-860.2)	182.8 (47.7-883.6)	227.5 (50.7-908.8)	0.485
Total bilirubin(μmol/L)	46.0 (19.1-174.7)	31.2 (18.9-165.3)	67.3 (19.5-198.0)	0.734
Tumor size (mm)	30 (20-40)	30 (20-40)	28 (20-40)	0.402
pT				0.286
T1/2	81 (68.1%)	28 (62.2%)	53 (71.6%)	
T3/4	38 (38.9%)	17 (37.8%)	21 (28.4%)	
pN				0.987
N0	66 (55.5%)	25 (55.6%)	41 (55.4%)	
N1	53 (44.5%)	20 (44.4%)	33 (44.6%)	
Differentiation				0.141
Well/Moderate	76 (63.9%)	25 (55.6%)	51 (68.9%)	
Poor	43 (36.1%)	20 (44.4%)	23 (31.1%)	
Nerve invasion				0.321
ne0	41 (34.5%)	18 (40%)	23 (31.1%)	
ne1,2,3	78 (65.5%)	27 (60%)	51 (68.9%)	
Location				0.236
Head	69 (58.0%)	23 (51.1%)	46 (62.2%)	
Neck, body, and tail	50 (42.0%)	22 (48.9%)	28 (37.8%)	

Data are presented as mean ± standard deviation, n (%), or median (25-75%).

CA19-9, Carbohydrate antigen-199.

**Figure 2:**
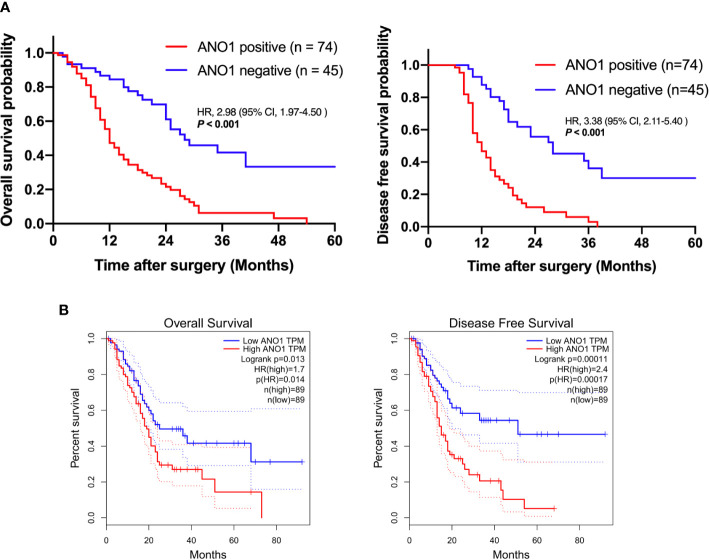
Survival analysis based on ANO1 expression. **(A)** Kaplan–Meier curves for overall survival and disease-free survival according to *ANO1* mRNA expression. **(B)** Kaplan–Meier curves for overall survival and disease-free survival according to ANO1 positivity. ANO1, aminooctylamine.

When using the immunohistochemistry results, Kaplan–Meier analysis showed that positive ANO1 expression was meaningfully associated with poor OS (log rank P < 0.001) and poor DFS (log rank P < 0.001) (**Figure 2A**). Similarly, in the TCGA dataset, high ANO1 mRNA levels were correlated with poor OS and DFS (**Figure 2B**).

Univariate and multivariate Cox regression analyses displayed that lymphatic metastasis (hazard ratio = 4.073, P = 0.001) and ANO1 expression (hazard ratio = 4.137, P = 0.001) were independent risk factors of the OS (**Table 2**).

**Table 2:** Univariate and multivariate analysis of overall survival according to ANO1 expression in patients with pancreatic cancer.

Characteristics	Univariate analysis		Multivariate analysis	
	HR	P	HR	P
**Age >65 years**	0.941	0.777		
**male**	0.692	0.092		
**CA19-9 >37 ng/mL**	1.530	0.108		
**Total bilirubin >1 μmol/L**	1.300	0.348		
**Tumor size >30 mm**	1.202	0.403		
**pT3,4**	1.004	0.984		
**pN1**	3.128	0.001	4.073	0.001
**Poor Differentiation**	0.727	0.142		
**Ne1,2,3**	0.740	0.163		
**Location (head)**	1.011	0.958		
**ANO1 positivity**	3.203	0.001	4.137	0.001

ANO1, aminooctylamine; HR, hazard ratio; CA19-9, Carbohydrate antigen-199.

### 3.3 The relationships between ANO1 expression and CAF subgroups

**Figure 3:**
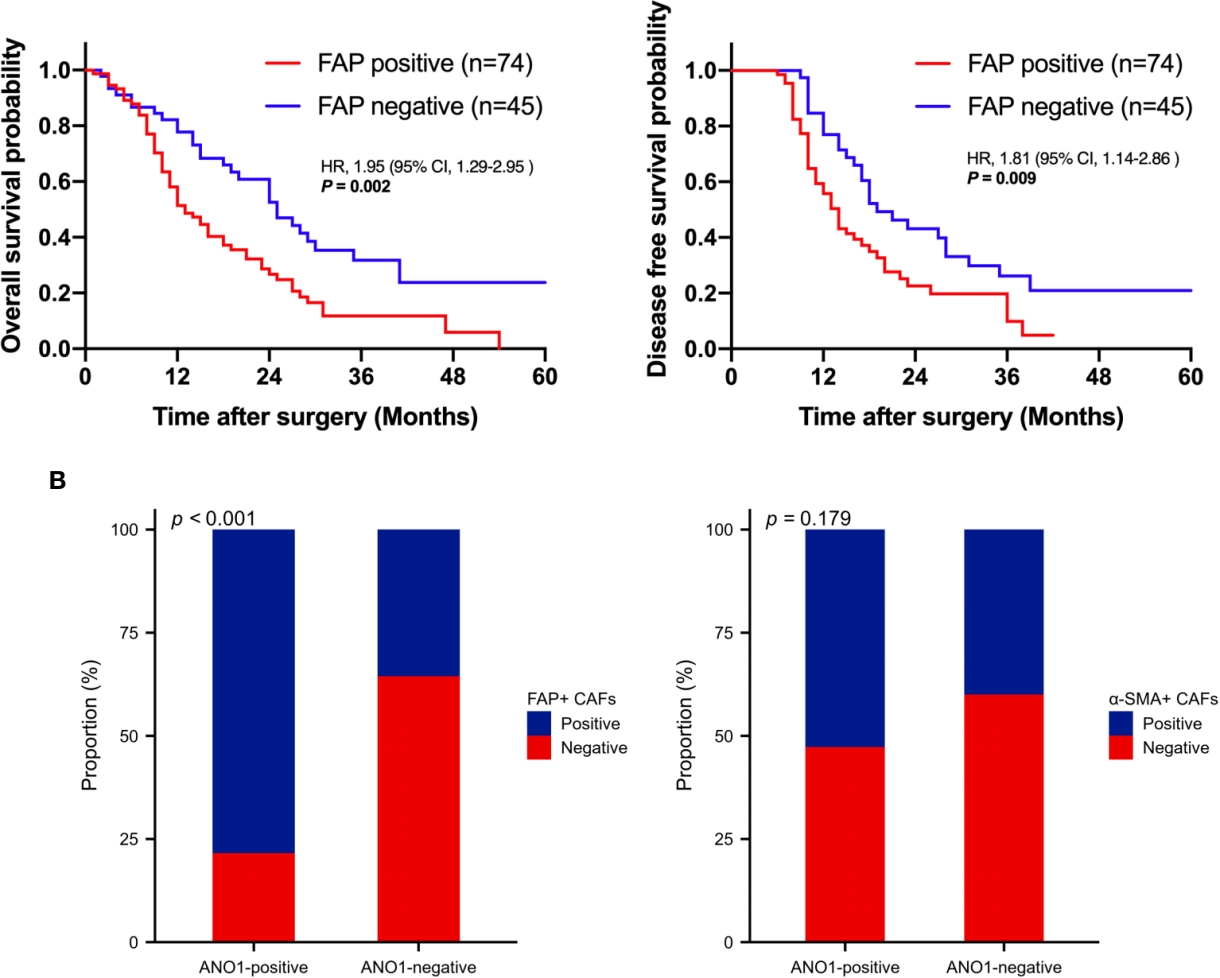
Survival analysis based on FAP positivity and correlation analysis between ANO1, FAP, and α-SMA. **(A)** Kaplan–Meier curves for overall survival and disease-free survival according to FAP positivity. **(B)** Correlation analysis between ANO1, FAP, and α-SMA. FAP, fibroblast activation protein; ANO1, aminooctylamine; α-SMA, alpha smooth muscle actin.

Kaplan–Meier analysis showed that positive FAP expression in CAFs was expressively associated with poor OS (log rank P = 0.002) and poor DFS (log rank P = 0.009), indicating that FAP-positive CAFs are associated with poor prognosis (**Figure 3A**). ANO1 expression was really correlated with FAP expression in CAFs (P < 0.001); however, ANO1 expression and α-SMA expression in CAFs had no virtual correlation (P = 0.179) (**Figure 3B**).

### 3.4 The relationship between ANO1 expression and TILs

**Figure 4:**
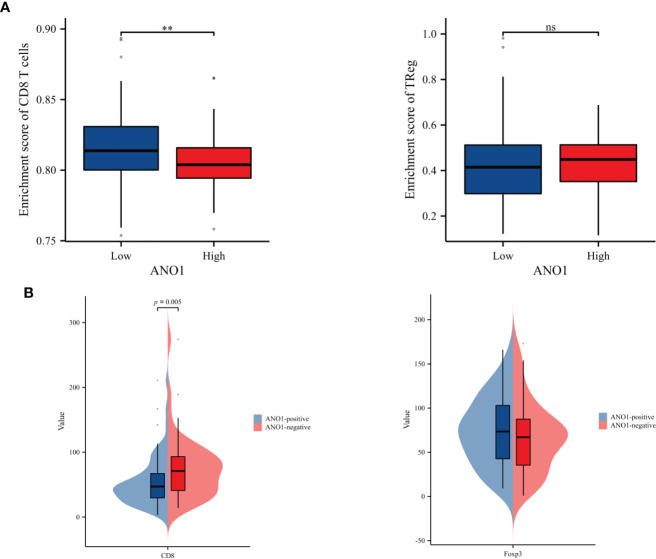
Correlation analysis between ANO1 and the numbers of CD8-positive and FOXP3-positive TILs. **(A)** The boxplot of the relationships between ANO1 and CD8 T cells and Tregs by ssGSEA. **(B)** Correlation analysis between ANO1 and the numbers of CD8-positive and FOXP3-positive TILs. ANO1, aminooctylamine; TIL, tumor-infiltrating lymphocyte; FOXP3, forkhead box protein 3; Treg, regulatory T cell; ssGSEA, single sample gene set enrichment analysis; **: p < 0.01; ns: not significant.

ssGSEA was performed to probe into the correlation between ANO1 expression and immune cell infiltration. The expression of ANO1 was inversely associated with the infiltration of CD8-positive TILs (P < 0.01) and had a potential relevance to FOXP3-positive TILs without a significant difference (**Figure 4A**). Interestingly, our IHC results were similar. ANO1 expression was inversely correlated with CD8-positive TILs (P = 0.005) and possibly positively correlated with FOXP3-positive TILs (**Figure 4B**).

### 3.5 ANO1 may induce an immunosuppressive tumor microenvironment in pancreatic cancer through a paracrine manner

**Figure 5:**
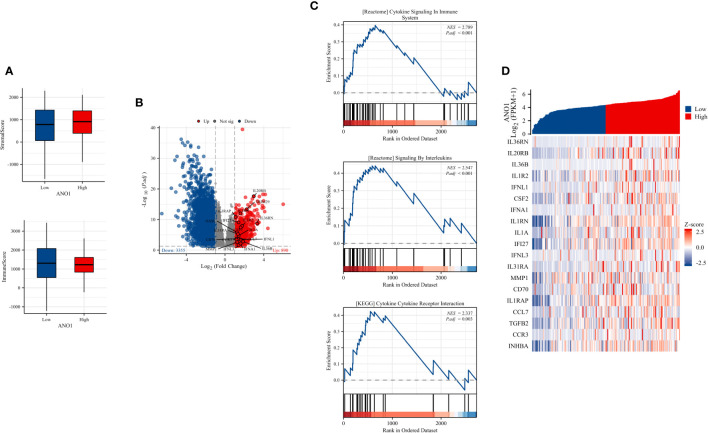
ImmuneScore, StromalScore, identiFIcation of DEGs based on ANO1 expression, and GSEA pathway enrichment. **(A)** The boxplot of the StromalScore and ImmuneScore of PC patients based on ANO1 expression. **(B)** The distribution of DEGs in the high and low ANO1 expression groups using volcano plots. **(C)** GSEA pathway enrichment analyses. **(D)** The heatmap of cytokines and receptors in the high and low ANO1 expression groups. DEG, Differential Expression Analysis; ANO1, aminooctylamine; GSEA, gene set enrichment analysis; PC, pancreatic cancer.

These results indicate that ANO1 may be highly correlated with an immunosuppressive tumor microenvironment in PC by interacting with FAP-positive CAFs and CD8-positive TILs. To further assess this, immune inFIltration (ImmuneScore) and total stromal content (StromalScore) were calculated using the ESTIMATE algorithm, and the results revealed that high ANO1 expression was correlated with a relative higher StromalScore and lower ImmuneScore (**Figure 5A**).

Bioinformatics analysis of the TCGA dataset was used to explore how ANO1 induces an immunosuppressive tumor microenvironment. We used the cutoff value that induced the most valid difference as the expression threshold to class the high and low ANO1 expression cohorts. In total, 890 genes were upward and 3355 genes were downward (**Figure 5B**). Furthermore, Reactome pathway enrichment analysis showed that “cytokine signaling in immune system” and “signaling by interleukins” were two possible pathways associated with ANO1, and KEGG pathway enrichment break down revealed that “cytokine-cytokine receptor interaction” was a possible pathway associated with ANO1 (**Figure 5C**). A heat map and **Table 3** showed that multiple cytokines and receptors were highly correlated with ANO1 expression (**Figure 5D**). In summary, the function of ANO1 in the TME may involve paracrine mechanisms.

**Table 3:** Correlation between ANO1 expression and cytokines and their receptors.

Name	Log2FoldChange	*P* value
IL36RN	3.316874325	2.00614E-09
IL20RB	2.965935052	2.4319E-18
IL36B	1.860432719	0.001536937
IL1R2	1.774740442	1.25503E-08
IFNL1	1.619699413	0.000355941
CSF2	1.614460098	3.973E-08
IFNA1	1.612978271	0.006911448
IL1RN	1.600195515	1.02219E-12
IL1A	1.418821194	1.82205E-05
IFI27	1.400404271	8.84722E-10
IFNL3	1.395986775	0.022168648
IL31RA	1.327320896	5.49237E-06
MMP1	1.255647561	0.002562962
CD70	1.172359866	0.000176168
IL1RAP	1.090599539	1.43772E-11
CCL7	1.354835717	0.000199447
TGFB2	1.260333078	6.35852E-08
CCR3	1.18356959	0.000365243
INHBA	1.087432619	9.39505E-06

## 4 Discussion

Multiple studies have identified the effects of ANO1 on tumor cells in various types of cancer,however, its effects on PC and the TME of PC have rarely been studied. Our results indicate that ANO1 positivity is an independent risk factor for PC. Further, ANO1 expression correlated positively with accumulation of FAP-positive CAFs. ANO1 high expression was significantly correlated with a lower number of CD8-positive TILs and possibly correlated with a high number of FOXP3-positive TILs. Because increased FAP-positive CAFs and decreased CD8-positive TILs are associated with an immunosuppressive environment in PC, ANO1 may be a key regulatory factor of the TME in PC. A bioinformatics analysis of the TCGA dataset showed the same results. Furthermore, the possible mechanism by which ANO1 affects the TME was investigated, and the results indicated a paracrine mechanism was involved. To the best of our knowledge, this study is the first to identify the prognostic role of ANO1 and its effect on the TME in PC.

The most specific hallmark of PC is remarkable desmoplasia/fibrosis, which accounts for 80% of the tumors and infiltrating immunosuppressive cells (28). CAFs, including activated pancreatic stellate cells, and their deposition in the extracellular matrix influence tumor progression, metastasis, therapeutic resistance, and angiogenesis (29). CAFs play a key role in tumor promotion through paracrine signaling and direct physical interactions (30). Recent lineage-tracing studies on pancreatic stellate cells have shown that native pancreatic fibroblast populations differ in their ability to expand during carcinogenesis (31, 32). CAFs with opposing functions in PC progression have been identified through single-cell RNA sequencing, multiple immunostaining, and various genetic mouse models (21). myCAFs and iCAFs play the most important functional roles and express FAP and α-SMA, respectively. A previous study showed that depletion of FAP-positive CAFs leads to increase survival rate, while the depletion of α-SMA-positive CAFs, which leads to decrease survival rate (16). PCs tend to have little CD8-positive T cell infiltration but are readily infiltrated by multiple types of immunosuppressive cells, such as FOXP3-positive Tregs, myeloid suppressor cells, and macrophages (33). previous studies have shown that increased numbers of CD8-positive TILs are associated with better survivalin patients with colorectal and ovarian cancer, whereas an increase in the number of Tregs is linked with poor survival in patients with PC (24, 34). Notably, CAFs have a clear relationship with immune cells. FAP-positive CAFs and α-SMA-positive CAFs can regulate both tumor-related pathways and Treg accumulation. Inhibition of α-SMA-positive CAFs can reduce the Teff/Treg ratio and promote tumor growth (35). Conversely, inhibition of FAP-positive CAFs decreases CD11b-positive myeloid cells and reduces PC growth (20, 36). Previous studies have shown that the general functional contribution of α-SMA-positive CAFs is tumor suppression, which is partly mediated by its production of type I collagen and IL-6 (37). However, this IL-6 production does not promote the progression of PC, and we observed an approximately 50% decrease in IL-6 in the TME (16). FAP-positive CAFs inhibit T cell infiltration into the tumor through CXCL12, and depletion of these fibroblasts or targeting of CXCL12 increases the sensitivity to immune checkpoint blockade of PC in preclinical models (38). Interestingly, although CD4-positive T cell ablation enables CD8-positive T cell-mediated deterioration of pancreatic tumors in mice, consumption of FOXP3-positive Tregs accelerates tumorigenesis as a result of compensatory myeloid infiltration (39). Our study found that ANO1 expression positively correlated with FAP-positive CAFs and negatively correlated with CD8-positive TILs, suggesting that ANO1 may be a key regulator of the tumor immune microenvironment.

Ion channel dysfunction is an emerging field in cancer research, and the differential expression of several types of ion channels in cancer have been reported (15, 26, 40). As a calcium-activated chloride channel, ANO1 can increase vascular smooth muscle contractility and can be a promising target for treating hypertension (41). The expression of ANO1 is influenced by multiple molecular mechanisms (42). Recent studies have revealed that ANO1 expression is controlled by 11q13 gene amplification and that ANO1 may play a cell-specific role through its interconnected protein network, phosphorylation of different kinases, and signaling pathways (11). We found that ANO1 is highly expressed in various malignancies and is associated with poor survival outcomes, which highlight its potential as a biomarker for the detection of certain malignancies. ANO1 not only inhibits tumor apoptosis but also promotes tumor immune escape. Recent researches have revealed that ANO1 overexpression promotes the progression of lung cancer, head and neck squamous cell carcinoma, and breast cancer through the epidermal growth factor receptor signaling pathway (43, 44). Specifically, in breast cancer cells, ANO1, epidermal growth factor receptor, and signal transducer and transcriptional activator 3 form a positive feedback pathway that promotes cancer cell proliferation and growth (45). ANO1 also activates the Ras-Raf-MEK-ERK1/2 signaling pathway, contributing to the development of head and neck squamous cell carcinoma and colon cancer (45). In esophageal squamous cell carcinoma, ANO1 regulates the tumor growth factor-B signaling pathway and promotes proliferation, migration, and invasion (46). In addition, ANO1 promotes metastasis of liver cancer and ovarian cancer by activating the phosphatidylinositol 3 kinase/protein kinase B signaling pathway (13, 47). However, the relationship between ANO1 and the TME has attracted increasing attention. Research have revealed that ANO1 is participate in immune escape in digestive system-related tumors (48). In one study, ANO1-expressing esophageal squamous cell carcinoma cells activated NFκB signaling in fibroblasts, stimulating CCL1 production, and subsequently enhance invasion (49). In gastrointestinal stromal tumors, the expression of ANO1 was significantly negatively correlated with infiltration of plasma cells and memory-activating CD4-positive T cells, suggesting that ANO1 may be participate in the functional suppression of T cells (50). ANO1 also conduces to immunosuppression of TME and induces acquired resistance to anti-PD-1 immunotherapy, while knockdown or inhibition of ANO1 enhances immunotherapy efficacy and overcomes resistance to immunotherapy. From the mechanism, ANO1 inhibits ferroptosis of cancer in a phosphatidylinositol 3 kinase/protein kinase B signaling-dependent manner, promotes tumor progression by promoting tumor growth factor-B release, and promotes the recruitment of CAFs, thereby anti-tumor immunity mediated by CD8-positive T cells is weakened and resistance to immunotherapy is generated (51). However, the role of ANO1 in PC has rarely been investigated. We speculated that ANO1 might regulate the TME through regulating the infiltration of FAP-positive CAFs and CD8-positive TILs in a paracrine manner. Through bioinformatics analysis of the TCGA dataset, we found that ANO1 may regulate the TME of PC through cytokine signaling, interleukin signaling, and cytokine-cytokine receptor interaction signaling pathways; however, the specific mechanisms remain to be further studied.

Our study has certain limitations. First. this was a single-center study, and the sample size limited the validity of our results. Therefore, a larger number of cases and mechanistic studies are required to validate our results. Second, to our knowledge, this is the first research to report that ANO1 is positively associated with CD8-positive TILs and FAP-positive CAFs in PC. However, our analysis used only immunohistochemistry, Therefore, this phenomenon needs to be further confirmed *in vivo* and *in vitro*.

In conclusion, ANO1 is an important prognostic factor for patients with PC after radical resection. ANO1 may induce an immunosuppressive tumor microenvironment in PC cells in a paracrine manner; therefore, ANO1 may be a new therapeutic target in PC.

## Data availability statement

The raw data supporting the conclusions of this article will be made available by the authors, without undue reservation.

## Ethics statement

The studies involving humans were approved by Institutional Review Committee of the Affiliated Hospital of North Sichuan Medical College (number: 2023ER041-1). The studies were conducted in accordance with the local legislation and institutional requirements. The human samples used in this study were acquired from primarily isolated as part of your previous study for which ethical approval was obtained. Written informed consent for participation was not required from the participants or the participants’ legal guardians/next of kin in accordance with the national legislation and institutional requirements.

## Author contributions

GZ: Conceptualization, Data curation, Methodology, Writing – original draft. ZS: Data curation, Formal analysis, Methodology, Writing – original draft. JY: Writing – original draft. JL: Writing – original draft. PY: Writing – original draft. BW: Writing – original draft. DD: Writing – original draft. SY: Writing – original draft. YL: Writing – original draft. DR: Writing – original draft. YH: Writing – review & editing. CL: Writing – original draft, Writing – review & editing.
